# Blood biomarker changes following therapeutic hypothermia in ischemic stroke

**DOI:** 10.1002/brb3.3230

**Published:** 2023-09-18

**Authors:** Elena Palà, Anna Penalba, Alejandro Bustamante, Teresa García‐Berrocoso, Marcel Lamana‐Vallverdú, Christian Meisel, Andreas Meisel, H. Bart van der Worp, Malcolm R Macleod, Bernd Kallmünzer, Stefan Schwab, Joan Montaner

**Affiliations:** ^1^ Neurovascular Research Laboratory Vall d'Hebron Institute of Research (VHIR)–Universitat Autónoma de Barcelona Barcelona Spain; ^2^ Stroke Unit, Hospital Universitari Germans Trias i Pujol Badalona Spain; ^3^ CSIC/UAB Proteomics Laboratory Institute of Biomedical Research of Barcelona Spanish National Research Council (IIBB‐CSIC/IDIBAPS) Barcelona Spain; ^4^ Institute for Medical Immunology Charité–Universitätsmedizin Berlin Berlin Germany; ^5^ Department of Immunology Labor Berlin–Charité Vivantes Berlin Germany; ^6^ Department of Neurology and Center for Stroke Research Berlin Charité University Hospital Berlin Berlin Germany; ^7^ Department of Neurology and Neurosurgery Brain Center University Medical Center Utrecht Utrecht The Netherlands; ^8^ Centre for Clinical Brain Sciences University of Edinburgh Edinburgh Scotland UK; ^9^ Department of Neurology Universitätsklinikum Erlangen Erlangen Germany; ^10^ Institute de Biomedicine of Seville, IBiS/Hospital Universitario Virgen del Rocío/CSIC/University of Seville & Department of Neurology Hospital Universitario Virgen Macarena Seville Spain

**Keywords:** biomarkers, hypothermia, ischemia, stroke

## Abstract

**Introduction:**

Therapeutic hypothermia is a promising candidate for stroke treatment although its efficacy has not yet been demonstrated in patients. Changes in blood molecules could act as surrogate markers to evaluate the efficacy and safety of therapeutic cooling.

**Methods:**

Blood samples from 54 patients included in the EuroHYP‐1 study (27 treated with hypothermia, and 27 controls) were obtained at baseline, 24 ± 2 h, and 72 ± 4 h. The levels of a panel of 27 biomarkers, including matrix metalloproteinases and cardiac and inflammatory markers, were measured.

**Results:**

Metalloproteinase‐3 (MMP‐3), fatty‐acid‐binding protein (FABP), and interleukin‐8 (IL‐8) increased over time in relation to the hypothermia treatment. Statistically significant correlations between the minimum temperature achieved by each patient in the hypothermia group and the MMP‐3 level measured at 72 h, FABP level measured at 24 h, and IL‐8 levels measured at 24 and 72 h were found. No differential biomarker levels were observed in patients with poor or favorable outcomes according to modified Rankin Scale scores.

**Conclusion:**

Although the exact roles of MMP3, FABP, and IL‐8 in hypothermia‐treated stroke patients are not known, further exploration is needed to confirm their roles in brain ischemia.

## Graphical abstract

Changes in blood molecules could act as surrogate markers to evaluate the efficacy and safety of therapeutic cooling. After evaluating a panel of 27 biomarkers including matrix metalloproteinases, cardiac, and inflammatory markers in the blood of patients treated with hypothermia and controls, MMP‐3, FABP, and IL‐8 increased over time in relation to the hypothermia treatment. These proteins deserve further exploration to confirm their role in brain ischemia.

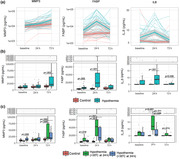

## 1 INTRODUCTION

Therapeutic hypothermia is a promising candidate for stroke treatment. Despite its benefits in preclinical studies, its efficacy has not yet been demonstrated in patients (Kuczynski et al., 2020). The EuroHYP‐1 study was stopped prematurely because it had a slow recruitment rate and was underpowered to detect clinically relevant differences regarding the outcome (van der Worp et al., 2019). However, changes in blood molecules could act as surrogate markers of efficacy and thus demonstrate the possible therapeutic effect of cooling. For instance, one of the mechanisms of action of hypothermia could be its action on the blood–brain barrier (BBB), reducing its damage (Kurisu & Yenari, 2019). Matrix metalloproteinases are among the most well‐known pathways involved in BBB leakage (Seo et al., 2011); therefore, the beneficial effects of hypothermia might be evaluated by analyzing the modulation of the components of this proteolytic family (e.g., matrix‐metalloproteinase‐2 (MMP‐2) and matrix metalloproteinase‐9 (MMP‐9); Jong et al., 2005). Additionally, some inflammatory and brain damage markers are well correlated with infarct growth and stroke outcomes (e.g., glial fibrillary acidic protein (GFAP), Amalia, 2021; D‐dimer, Zhang et al., 2021; brain derived neurotrophic factor (BDNF), Mojtabavi et al., 2022; and interleukin‐6 (IL‐6), Bustamante et al., 2014); therefore, members of these families could serve as ideal candidate biomarkers to reflect hypothermia biological responses.

In addition, other molecules could be used as markers of safety, identifying possible hypothermia‐related adverse effects in ischemic stroke patients, such as infections or cardiac complications. For this purpose, previously defined immune system‐related biomarkers (e.g., interleukin‐8 (IL‐8), interleukin‐10 (IL‐10), lipopolysaccharide binding protein (LBP), mannose‐binding lectin (MBL), procalcitonin, and copeptin) and stress‐related molecules (e.g., mid‐regional pro‐atrial natriuretic peptide (MR‐proaANP) and N‐terminal pro‐brain natriuretic peptide (NT‐proBNP)) could be of interest.

In the present study, we hypothesized that the study of circulating proteins may provide a “proof of concept” of hypothermia and elucidate mechanisms associated with therapeutic cooling that may help in the design of future trials. Therefore, and following the above reasoning, we selected BBB, inflammatory, brain damage, and cardiac damage markers to assess the effect of hypothermia on these pathways.

## 2 METHODS

### 2.1 Study population

The EuroHYP‐1 study was a randomized multicenter clinical trial comparing the efficacy and safety of therapeutic hypothermia after acute ischemic stroke versus best medical treatment (BMT). Details on the inclusion and exclusion criteria and the study protocol can be found elsewhere (van der Worp et al., 2014, 2019). Briefly, included patients were allocated to receive hypothermia plus standard care or to standard care alone. In patients treated with hypothermia, cooling was started within 6 h after the onset of symptoms and within 150 min of the start of thrombolysis, endovascular treatment, or hospital admission in patients not receiving any of these. Cooling was maintained at 34−35°C for 12–24 h (the planned duration of active cooling was reduced from 24 to 12 h after the inclusion of the first 50 patients into the trial to increase the feasibility; Supplemental Figure S1). The choice of the cooling technique was at the discretion of the local investigator as long as protocol‐specified devices were used. Surface or endovascular methods were accepted (i.e., a Medivance/Bard Arctic Sun temperature management system, MTRE CritiCool temperature management system, or Zoll intravascular temperature management system). Thereafter, patients were passively rewarmed at a rate of 0.2–0.1°C per hour until the rectal or bladder temperature reached 36°C.

Ethics approval was obtained in each country and (where required) at each site before the start of the study. All patients or their representatives provided written informed consent. The trial was conducted in accordance with the ethical principles that have their origin in the Declaration of Helsinki and are consistent with the International Conference on Harmonization‐Good Clinical Practice and applicable regulatory requirements. The trial was registered at ClinicalTrials.gov as NCT01833312.

### 2.2 Biomarker measurement

Blood samples were obtained at three time points; baseline, 24 ± 2 h, and 72 ± 4 h after the initiation of the hypothermia treatment (Supplemental Figure S1). Blood was collected into EDTA‐ and serum‐containing tubes and centrifuged at 1500 × g for 15 min at 4°C, and aliquots were frozen at −80°C until biomarker measurement. The blood collection and material analysis protocols were standardized among centers.

Twenty‐seven biomarkers in all the samples were measured centrally at Vall d'Hebron Institute of Research (Barcelona, Spain; matrix metalloproteinase‐1 (MMP‐1), MMP‐2, matrix metalloproteinase‐3 (MMP‐3), matrix metalloproteinase‐7 (MMP‐7), MMP‐9, matrix metalloproteinase‐10 (MMP‐10), matrix metalloproteinase‐12 (MMP‐12), matrix‐metalloproteinase‐13 (MMP‐13), TIMP metalloproteinase inhibitor 1 (TIMP‐1), and TIMP metalloproteinase inhibitor 2 (TIMP‐2)), Randox Laboratories (Crumlin, UK; BDNF, C‐reactive protein (CRP), D‐dimer, FABP, GFAP, IL‐6, neutrophil gelatinase‐associated lipocalin (NGAL), neuron‐specific enolase (NSE), and tumor necrosis factor receptor 1 (TNFR‐1)), and Charité Universitätsmedizin Berlin (Germany; NT‐proBNP, Copeptin, MR‐proANP, procalcitonin, IL‐8, LBP, IL‐10, and MBL) by the immunoassay techniques listed in Supplemental Table S1.

### 2.3 Statistical analyses

The statistical analyses were conducted with SPSS version 20 and R version 4.04. Categorical variables are expressed as numbers and percentages. The distribution of continuous variables was assessed using the Kolmogorov–Smirnov test. Continuous variables were expressed as the mean ± standard deviation for normally distributed data and median (interquartile range) for nonnormally distributed data. For univariate analysis, continuous variables were compared by the Mann–Whitney U‐test or Student's *t*‐test depending on variable distribution, and categorical variables were compared by the χ^2^ test. The Spearman coefficient was used to test correlations.

Samples below and above the limit of detection were substituted by the most extreme values detected in the same experiment for the analysis (Supplemental Table S1). Missing biomarker data were imputed with the mice R package (cart methodology). Then, generalized square models with each biomarker logarithmically transformed as an endpoint were fitted. The baseline time was considered a covariate, and the effect of time and treatment was explored using the following formula: log(biomarker) ∼ log(biomarker_baseline) + time * treatment. The effects of the hypothermia treatment on the circulating levels of the proteins tested over time were evaluated by the presence of a statistically significant hypothermia * time interaction term with ANOVA using the generalized square models described above. To correct for the role of infections in the biomarker levels, a secondary analysis was performed by adding the infections to the models as covariates. Bonferroni correction was used to correct for multiple comparisons, and an adjusted *p*‐value < .05 was considered statistically significant.

Finally, association with clinical outcome was tested in those biomarkers in which a statistically significant hypothermia * time interaction term was found. To do so, patients were classified according to the modified Rankin Scale (mRS) scores as having poor (mRS score > 2) or favorable outcomes (mRS score≤2), and differences in biomarker levels were tested by the Mann–Whitney U‐test. Analyses were performed including all patients and separating them by treatment.

## 3 RESULTS

Samples from 54 patients were available (27 treated arm and 27 control arm). Of these, 43 patients had a complete temporal profile. Samples from five patients at baseline, one at 24 h and seven at 72 h could not be collected for several reasons. In total, 149 samples were available. The baseline characteristics of patients receiving the treatment and controls were similar (Supplemental Table S2). Of the 27 patients randomized to receive hypothermia treatment, nine were still in hypothermia (<35°C) when the second blood sample was collected (24 ± 2 h after the initiation of the treatment).

**FIGURE 1:**
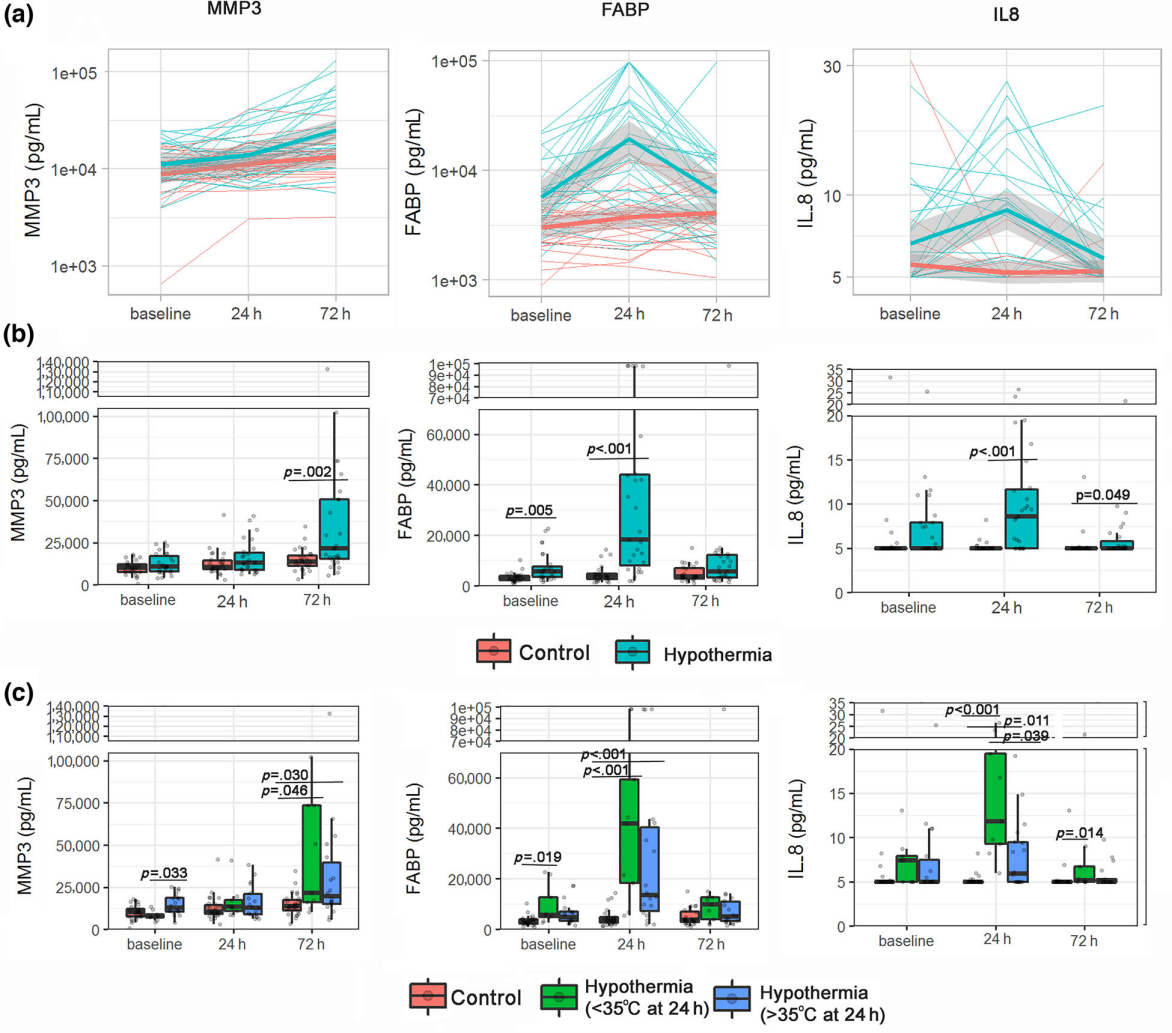
Longitudinal blood biomarker changes associated with hypothermia therapy in stroke patients. (a) The biomarker levels and the temporal profile of each patient are shown with fine lines. Additionally, a trend line describing the general pattern of the data for controls (red) and hypothermia patients (blue) is drawn. (b) The distribution of the three proteins in the entire cohort is shown, classifying the patients according to the treatment received. (c) The boxplot distribution of the biomarkers separates the individuals treated with hypothermia depending on the hypothermia status at 24 h (> or < 35°C). Statistical differences at a specific time point are indicated. Boxes extend from the 25th to the 75th percentiles. The line in the middle is plotted as the median. Whiskers are drawn according to the Tukey methodology (±1.5 interquartile range).

MMP‐3, FABP, and IL‐8 changed over time in relation to the hypothermia treatment when adjusted by multiple comparisons (Table 1). MMP‐3 increased with a steeper slope after initiation of the treatment in the hypothermia group in comparison to the control group, showing higher levels in the treated group after 72 h. FABP and IL‐8 rose in the hypothermia group after the treatment phase, lowering their levels after 72 h, while in the control group, levels did not change (Figure 1a,b). Patients still in hypothermia at 24 h had higher levels of IL‐8 than patients who were already rewarmed, while the other two biomarkers showed similar results (Figure 1c). The addition of infections into the model did not influence the obtained results (data not shown).

**TABLE 1:** Effects of hypothermia treatment on circulating levels of the proteins tested over time.

Biomarker	*p*‐value Intervention: time	*p*‐value adjusted
MMP‐1	.568	1.00
MMP‐2	.0369	.9963
MMP‐3	.0018	.0486
MMP‐7	.2435	1.00
MMP‐9	.0270	.729
MMP‐10	.0027	.0729
MMP‐12	.3045	1.00
MMP‐13	.2072	1.00
TIMP‐1	.0390	1.00
TIMP‐2	.7871	1.00
BDNF	.4913	1.00
CRP	.2690	1.00
D‐dimer	.1314	1.00
FABP	.00006	.00162
GFAP	.6640	1.00
IL‐6	.0100	.27
NGAL	.0888	1.00
NSE	.5639	1.00
TNFR‐1	.1715	1.00
NT‐proBNP	.7871	1.00
Copeptin	.0022	.0594
MR‐proANP	.3878	1.00
Procalcitonin	.6789	1.00
IL‐8	.0011	.0297
LBP	.2590	1.00
IL‐10	.0675	1.00
MBL	.6163	1.00

Statistically significant correlations between the minimum temperature achieved by each patient in the hypothermia group and the MMP‐3 level measured at 72 h (*r* = −.477; *p* = .021), FABP level measured at 24 h (*r* = −.408; *p* = .043), and IL‐8 levels measured at 24 h (*r* = −.585; *p* = .002) and 72 h (*r* = −.605; *p* = .002) were found (Supplemental Figure S2).

No differential biomarker levels were observed in patients with poor or favorable outcomes according to mRS scores, except an increase in FABP at 72 h in the patients with poor outcomes, in the control group (*p* = .033).

## 4 DISCUSSION

Few studies have explored the changes in circulating molecules in relation to hypothermia treatment after stroke (Ávila‐Gómez et al., 2020; Horstmann et al., 2003). In the present study, we evaluated circulating levels of biomarkers in relation to possible beneficial mechanisms and side effects of cooling at three time points: before, during hypothermia/rewarming, and after rewarming. However, contrary to what we expected, we did not observe a decrease in matrix metalloproteases or inflammatory markers, proteins typically increased after the acute phase of stroke (Montaner et al., 2019; Simats et al., 2016), a decrease that could have been indicative of the beneficial effect of hypothermia. In contrast, once corrected by the baseline levels, we reported an increase in some proteins in the hypothermia group, such as MMP‐3, FABP, and IL‐8.

MMP‐3, similar to other metalloproteinases, is a proteolytic enzyme that degrades protein constituents of the extracellular matrix, but it also contributes to other cell signaling pathways (Van Hove et al., 2012). In the acute phase of ischemic stroke, the expression of this protein has been reported, playing a key role in the degradation of tight junction proteins and BBB disruption (Montaner et al., 2019). Only a few studies have tested the circulating levels of MMP‐3 after stroke. A small study assessing therapeutic hypothermia indicated that this protein increased in the hyperacute phase of stroke but gradually recovered to control levels after the first hour. However, the authors did not show any effect of hypothermia on MMP‐3 levels, but only 15 patients were included (Horstmann et al., 2003). Apart from the pathological role of metalloproteinases in stroke, emerging evidence has pointed out their importance in tissue regeneration processes, including poststroke repair. In fact, higher MMP‐3 blood levels were associated with a better neurological status in a small cohort of patients under rehabilitation (Ma et al., 2016). Interestingly, the temporal profile of MMP3, with an increase at 72 h, could be explained by a late consequence of the hypothermia or due to the effect of rewarming. The fact that MMP‐3 levels at 72 h correlated with the minimum temperature reached might support the first hypothesis.

FABP is a small cytoplasmic protein whose primary function is to transport long‐chain fatty acids, and it was first described as a diagnostic tool for heart conditions. Although it has been studied as a potential brain injury biomarker for several neurological disorders, including stroke, its exact role in stroke pathophysiology is still unknown (Lescuyer et al., 2005). Interestingly, FABP has been related to thermogenesis regulation and cold tolerance through its role in fatty acid oxidation in adipose tissue (Vergnes et al., 2011), which could explain the increase we observed under hypothermia and its correlation with the minimum temperature achieved. Additionally, in a study applying therapeutic hypothermia during cardiac surgery, the authors reported an elevation of FABP with a profile similar to the one we found (Rosenthal et al., 2020). However, the authors suggested that this was due to myocardial damage, but in our study, we did not observe any change in other cardiac‐related markers, such as NT‐proBNP or MR‐proANP. In the present study, an increase in FABP was shown in control patients with poor outcome at 72 h, but this cannot explain its increase after hypothermia, as this association was not seen in hypothermia patients.

Finally, IL‐8, together with other proinflammatory markers, is elevated as part of the ischemic cascade and has been associated with worse outcomes after ischemic stroke (Simats et al., 2016; Shaheen et al., 2018). It should be noted that IL‐8 increased in patients still in hypothermia (who showed even higher levels for extended periods) and correlated with the minimum temperature reached. Therefore, it seems that being in hypothermia is what triggers the expression of these molecules. Interestingly, in a clinical trial testing therapeutic hypothermia for the treatment of neonatal hypoxic ischemic encephalopathy, an increase in IL‐8 and other cytokines was also found in the treated group. This increase was negatively correlated with leukocyte counts in hypothermia patients (Jenkins et al., 2013). This could explain the increased risk of infections in the treated group found in the EuroHYP‐1 study, although information on leukocyte counts was not available (van der Worp et al., 2014). Nevertheless, it should be noted that in the present study, adding the infections as a covariate did not change the obtained results, and, therefore, the presence of infections alone could not explain the elevation of IL‐8. Alternatively, we cannot rule out the hypothesis that neuroinflammatory responses are not entirely harmful and that they may be part of an important mechanism for neuroprotection at certain time points (Ceulemans et al., 2010).

It should be noted that we do not know whether the changes in these biomarkers originate in the brain or are due to a systemic effect of hypothermia. In the future, the isolation of neural‐specific extracellular vesicles/exosomes could be performed, especially in local hypothermia studies (Liddle et al., 2022). Additionally, the usefulness of monitoring some of these or other biomarkers during the early time points of hypothermia (e.g., during the first 24 h) to guide the clinical pivot to additional rescue therapies should also be investigated. Nevertheless, in light of the present results, we do not see any clear relationship between the levels of the tested biomarkers and clinical/safety outcomes.

Finally, the methodology used in the present study does not allow us to draw conclusions on which are the main pathways of action that lead to the observed changes. However, it would be interesting to investigate, with further experiments and studies, the molecular mechanisms of the proteins presenting significant between‐group differences in response to hypothermia. In particular, it would be interesting to explore the role of neuroinflammation after hypothermia, as the three proteins showing changes could be classified as neuroinflammatory mediators, and it has been previously noted that hypothermia influences the expression of several inflammatory parameters, without clear conclusions on its negative or positive effects to neuroprotection (Ceulemans et al., 2010).

In summary, although the exact roles of MMP3, FABP, and IL‐8 in hypothermia‐treated stroke patients are not known, these proteins should be explored in new studies testing systemic and also selective hypothermia.

## 5 LIMITATIONS

We should acknowledge that low recruitment in the EuroHYP‐1 trial limited the sample size of the present study. Additionally, differences regarding the cooling active period among patients, with patients randomized to hypothermia not reaching the predefined cooling targets, could have influenced some of our results, especially leading to false‐negative results. Additionally, because some of the molecules tested can undergo large changes in concentration a few hours after the ischemic event, differences in the time elapsed since symptom onset could have influenced some biomarker levels. However, to control this limitation, we have corrected all the analyses by the baseline biomarker concentrations.

## AUTHOR CONTRIBUTIONS

H. Bart van der Worp, Malcolm R Macleod, Stefan Schwab, and Joan Montaner contributed to the conception and design of the study. Elena Palà, Anna Penalba, Alejandro Bustamante, Teresa García‐Berrocoso, Marcel Lamana‐Vallverdú, Bernd Kallmünzer, Christian Meisel, and, Andreas Meisel contributed to the acquisition and analysis of the data. Elena Palà contributed to drafting the text and preparing the figures. A detailed list of authors and affiliations of the EuroHYP‐1 group is provided in Supplemental Table S3.

### PEER REVIEW

The peer review history for this article is available at https://publons.com/publon/10.1002/brb3.3230.

## Supporting information

SUPPLEMENTAL TABLE S1 Methods of biomarker measurement.
SUPPLEMENTAL TABLE S2 Baseline characteristics of the study population.
SUPPLEMENTAL FIGURE S1 Schematic time line of the study.
SUPPLEMENTAL FIGURE S2 Correlations between MMP‐3, FABP, and IL‐8 concentrations at 24 and 72 h and the minimum temperature reached in patients treated with hypothermia.

SUPPLEMENTAL TABLE S3 EuroHYP group.

## Data Availability

The data that support the findings of this study are available from the corresponding author upon reasonable request.
