# Supplementary material for: Blood biomarker changes following therapeutic hypothermia in ischemic stroke
Source: Brain Behav. 2023 Sep 18;13(11):e3230. doi: 10.1002/brb3.3230 (PMC10636403; doi:10.1002/brb3.3230)
Supplement: Supplementary file 1 — SUPPLEMENTAL TABLE S1 Methods of biomarker measurement. SUPPLEMENTAL TABLE S2 Baseline characteristics of the study population. SUPPLEMENTAL FIGURE S1 Schematic time line of the study. SUPPLEMENTAL FIGURE S2 Correlations between MMP‐3, FABP, and IL‐8 concentrations at 24 and 72 h and the minimum temperature reached in patients treated with hypothermia. [file BRB3-13-e3230-s002.docx]

Supplemental Table 1: Methods of biomarker measurement.

| Biomarker | Manufacturer | Catalogue number | Matrix type | Detection range | Units | Num of samples below the detection range* | Num of samples above the detection range* |
| --- | --- | --- | --- | --- | --- | --- | --- |
| MMP-1 | Millipore | HMMP2MAG-55K | Plasma | 27- 20000 | pg/mL | 0 | 0 |
| MMP-2 | Millipore | MMP2MAG-55K | Plasma | 68- 50000 | pg/mL | 0 | 0 |
| MMP-3 | Millipore | HMMP1MAG-55K | Plasma | 146-150000 | pg/mL | 0 | 0 |
| MMP-7 | Millipore | HMMP2MAG-55K | Plasma | 548- 400000 | pg/mL | 0 | 0 |
| MMP-9 | Millipore | HMMP2MAG-55K | Plasma | 14-10000 | pg/mL | 0 | 0 |
| MMP-10 | Millipore | HMMP2MAG-55K | Plasma | 27- 20000 | pg/mL | 0 | 0 |
| MMP-12 | Millipore | HMMP1MAG-55K | Plasma | 98- 100000 | pg/mL | 0 | 0 |
| MMP-13 | Millipore | HMMP1MAG-55K | Plasma | 58- 60000 | pg/mL | 0 | 0 |
| TIMP-1 | Millipore | HTMP1MAG-54K | Plasma | 20-20000 | pg/mL | 0 | 0 |
| TIMP-2 | Millipore | HTMP1MAG-54K | Plasma | 49-50000 | pg/mL | 0 | 0 |
| BDNF | Randox | Cerebral Array I  EV3573 | Serum | 0 – 7500 | pg/mL | 0 | 0 |
| CRP | Randox | Cerebral Array I  EV3573 | Serum | 0-12 | mg/L | 0 | 15 |
| D-dimer | Randox | Cerebral Array I  EV3573 | Serum | 0-2000 | ng/mL | 0 | 41 |
| FABP | Randox | Cerebral Array I  EV3573 | Serum | 0 – 100 | ng/mL | 0 | 4 |
| GFAP | Randox | Cerebral Array I  EV3573 | Serum | 0 – 120 | ng/mL | 94 | 0 |
| IL-6 | Randox | Cerebral Array I  EV3573 | Serum | 0 – 550 | pg/ml | 0 | 0 |
| NGAL | Randox | Cerebral Array II  EV3637 | Serum | 0-2000 | ng/mL | 0 | 6 |
| NSE | Randox | Cerebral Array II  EV3637 | Serum | 0-200 | ng/mL | 0 | 1 |
| TNFR-1 | Randox | Cerebral Array II  EV3637 | Serum | 0-50 | ng/mL | 0 | 0 |
| NT-proBNP | Roche Diagnostics | 09315284190 | Serum | 5-3500 | ng/L | 0 | 0 |
| Copeptin | Brahms ThermoFisher | 857.050 | Serum | 0,7 - 500 | pmol/L | 0 | 0 |
| MR-proANP | Brahms ThermoFisher | 819.050 | Serum | 2,1 - 1000 | pmol/L | 0 | 0 |
| Procalcitonin | Roche Diagnostics | 08828679190 | Serum | 0,02 - 100 | µg/L | 44 | 0 |
| IL-8 | Siemens Healthineers | LK8P1 | Serum | 2 - 7500 | pg/mL | 92 | 0 |
| LBP | Siemens Healthineers | LKLB1 | Serum | 1,2 - 200 | µg/mL | 0 | 0 |
| IL-10 | Siemens Healthineers | LKXP1 | Serum | 1 - 1000 | pg/mL | 93 | 0 |
| MBL | Bioporto | KIT 029 | Serum | 50-4000 | ng/mL | 17 | 20 |

MMP-1: matrix metalloproteinase-1; MMP-2: matrix metalloproteinase-2; MMP-3: matrix metalloproteinase-3; MMP-7: matrix metalloproteinase-7; MMP-9: matrix metalloproteinase-9; MMP-10: matrix metalloproteinase-10; MMP-12: matrix metalloproteinase-12; MMP-13: matrix metalloproteinase-13; TIMP-1: TIMP metalloproteinase inhibitor 1; TIMP-2: TIMP metalloproteinase inhibitor 2; BDNF: brain-derived neurotrophic factor; CRP: C-reactive protein; FABP: fatty-acid-binding proteins; GFAP: glial fibrillary acidic protein; IL-6: interleukin-6; NGAL: neutrophil gelatinase-associated lipocalin; NSE: neuron-specific enolase; TNFR-1: tumor necrosis factor receptor 1; NT-proBNP: N-terminal pro-brain natriuretic peptide; MR-proANP: mid-regional pro-atrial natriuretic peptide; IL-8: Interleukin-8; LBP: lipopolysaccharide binding protein; IL-10: interleukin-10; MBL: mannose-binding lectin

*149 samples were available to be tested.

Supplemental Table 2: Baseline characteristics of the study population.

|  | Total (n=54) | Controls (n=27) | Hypothermia (n=27) | P-value |
| --- | --- | --- | --- | --- |
| Age | 75.5 (70-80) | 77 (71.5-80) | 72 (68-79) | 0.209 |
| Sex (%female) | 22 (40.7%) | 13 (48.1%) | 9 (33.3%) | 0.268 |
| Hypertension | 31 (68.9%) | 16 (76.2%) | 15 (62.5%) | 0.322 |
| Diabetes | 16 (35.6%) | 7 (33.3%) | 9 (37.5%) | 0.771 |
| Dyslipidemia | 9 (20.0%) | 6 (28.6%) | 3 (12.5%) | 0.179 |
| Previous stroke | 13 (28.9%) | 7 (33.3%) | 6 (25.0%) | 0.538 |
| Atrial fibrillation | 4 (8.9%) | 2 (9.5%) | 2 (8.3%) | 0.889 |
| Pneumonia | 6 (11.1%) | 2 (7.4%) | 4 (14.8%) | 0.386 |
| Infection | 11 (20.4%) | 3 (11.1%) | 8 (29.6%) | 0.091 |
| NIHSS baseline | 10 (7.75-16.25) | 9 (8-16) | 10 (7-17) | 0.965 |
| Thrombolysis | 45 (83.3%) | 23 (85.2%) | 22 (81.5%) | 0.715 |
| mRS at 90 days>2 | 30 (57.7%) | 17 (65.4%) | 13 (50.0%) | 0.262 |

NIHSS: National Institute of Health Stroke Scale; mRS:modified rankin scale

Supplemental Figure 1: Schematic time line of the study.


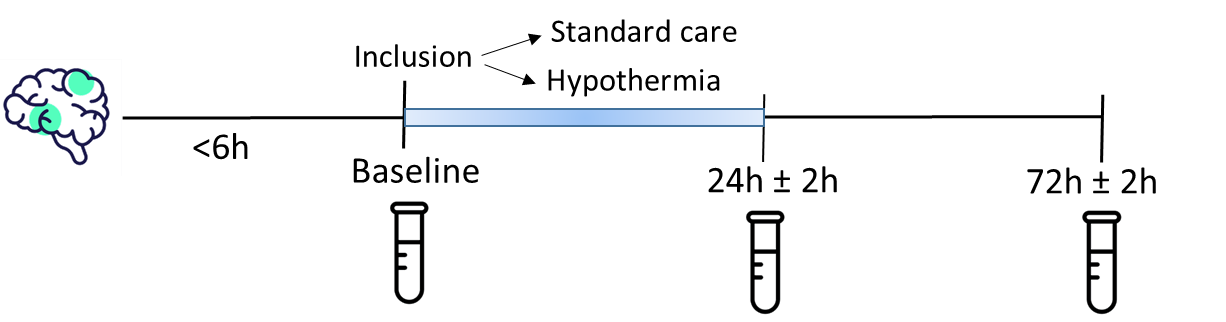


Patients were included within 6h after onset of symptoms and allocated to standard care or hypothermia. Cooling was maintained for 12-24 h. Blood samples were obtained at three time-points; baseline, 24 h ± 2 h, and 72 h ± 4 h since the initiation of the hypothermia treatment.

Supplemental Figure 2: Correlations between MMP-3, FABP, and IL-8 concentrations at 24h and 72h and the minimum temperature reached in patients treated with hypothermia.
