# Supplementary material for: Blood biomarker changes following therapeutic hypothermia in ischemic stroke
Source: Brain Behav. 2023 Sep 18;13(11):e3230. doi: 10.1002/brb3.3230 (PMC10636403; doi:10.1002/brb3.3230)
Supplement: Supplementary file 2 — SUPPLEMENTAL TABLE S3 EuroHYP group. [file BRB3-13-e3230-s001.docx]

Supplemental Table 3: Eurohyp group

| Name | Affiliation | Mail |
| --- | --- | --- |
| H Bart van der Worp | Department of Neurology and Neurosurgery, Brain Center, University Medical Center Utrecht, Utrecht, The Netherlands | H.B.vanderWorp@umcutrecht.nl |
| Malcolm R Macleod | Centre for Clinical Brain Sciences, University of Edinburgh, Edinburgh, Scotland, UK 3 4 | [Malcolm.Macleod@ed.ac.uk](mailto:Malcolm.Macleod@ed.ac.uk) |
| Philip MW Bath | Stroke Trials Unit, Division of Clinical Neuroscience, University of Nottingham, Nottingham, UK | [philip.bath@nottingham.ac.uk](mailto:philip.bath@nottingham.ac.uk) |
| Raj Bathula | Stroke Department, Northwick Park Hospital, London, UK | [r.bathula@nhs.net](mailto:r.bathula@nhs.net) |
| Hanne Christensen | Department of Neurology, Bispebjerg og Frederiksberg Hospitaler, University of Copenhagen, Copenhagen, Denmark | [hanne.krarup.christensen@regionh.dk](mailto:hanne.krarup.christensen@regionh.dk) |
| Bridget Colam | Centre for Clinical Brain Sciences, University of Edinburgh, Edinburgh, Scotland, UK 3 Stroke Trials Unit, Division of Clinical Neuroscience, University of Nottingham, Nottingham, UK | [Bridget.Colam@ed.ac.uk](mailto:Bridget.Colam@ed.ac.uk) |
| Charlotte Cordonnier | University of Lille, Inserm U1171, Degenerative and Vascular Cognitive Disorders, Centre Hospitalier Universitaire Lille, Lille, France | [charlotte.cordonnier@chru-lille.fr](mailto:charlotte.cordonnier@chru-lille.fr) |
| Jacques Demotes-Mainard | ECRIN, Paris, France | [jacques.demotes@ecrin.org](mailto:jacques.demotes@ecrin.org) |
| Isabelle Durand-Zaleski | APHP URCEco, University Paris Est Creteil & INSERM UMR 1123, Paris, France | [isabelle.durand-zaleski-ext@aphp.fr](mailto:isabelle.durand-zaleski-ext@aphp.fr) |
| Christian Gluud | Copenhagen Trial Unit, Centre for Clinical Intervention Research, Rigshospitalet, Copenhagen University Hospital, Copenhagen, Denmark | christian.gluud@ctu.dk |
| Janus Christian Jakobsen | Copenhagen Trial Unit, Centre for Clinical Intervention Research, Rigshospitalet, Copenhagen University Hospital, Copenhagen, Denmark.  Department of Cardiology, Holbæk Hospital, Copenhagen, Denmark. | [janus.jakobsen@ctu.dk](mailto:janus.jakobsen@ctu.dk) |
| Bernd Kallmünzer | Department of Neurology, University Medical Centre Erlangen, Erlangen, Germany | Bernd.Kallmuenzer@uk-erlangen.de |
| Rainer Kollmar | Klinik für Neurologie und Neurointensivmedizin, Klinikum Darmstadt, Darmstadt, Germany | [Rainer.Kollmar@mail.klinikum-darmstadt.de](mailto:Rainer.Kollmar@mail.klinikum-darmstadt.de) |
| Derk W Krieger | Mediclinic Middle East, Dubai, UAE | derk.krieger@gmail.com |
| Kennedy R Lees | School of Medicine, Dentistry & Nursing, University of Glasgow, Glasgow, UK | [kennedy.lees@glasgow.ac.uk](mailto:kennedy.lees@glasgow.ac.uk) |
| Dominik Michalski | Department of Neurology, University of Leipzig, Leipzig, Germany | [Dominik.Michalski@medizin.uni-leipzig.de](mailto:Dominik.Michalski@medizin.uni-leipzig.de) |
| Carlos Molina | Hospital Universitari Vall d’Hebron, Barcelona, Spain | [cmolina@vhebron.net](mailto:cmolina@vhebron.net) |
| Risto O Roine | Division of Clinical Neurosciences, Turku University Hospital and University of Turku, Turku, Finland | [risto.roine@tyks.fi](mailto:risto.roine@tyks.fi) |
| Jesper Petersson | Department of Neurology, Skane University Hospital, Malmo, Sweden | [jesper.petersson@skane.se](mailto:jesper.petersson@skane.se) |
| Richard Perry | Stroke Service, National Hospital for Neurology & Neurosurgery, Queen Square, London, UK | richard.perry2@nhs.net |
| Nikola Sprigg | Stroke Trials Unit, Division of Clinical Neuroscience, University of Nottingham, Nottingham, UK | nikola.sprigg@nottingham.ac.uk |
| Dimitre Staykov | Department of Neurology, Hospital of the Brothers of St. John, Eisenstadt, Austria.  Department of Neurology, University Medical Centre Erlangen, Erlangen, Germany | [staykov@gmx.at](mailto:staykov@gmx.at) |
| Istvan Szabo | European Stroke Research Network for Hypothermia, Brussels, Belgium | [istvan.szabo@esrnh.org](mailto:istvan.szabo@esrnh.org) |
| Geert Vanhooren | AZ Sint-Jan Brugge-Oostende, Brugge, Belgium AV | [geert@vanhooren-dooms.be](mailto:geert@vanhooren-dooms.be) |
| Joanna M Wardlaw | Edinburgh Imaging, Centre for Clinical Brain Sciences and UK Dementia Research Institute, University of Edinburgh, Edinburgh, UK | [joanna.wardlaw@ed.ac.uk](mailto:joanna.wardlaw@ed.ac.uk) |
| Per Winkel | Copenhagen Trial Unit, Centre for Clinical Intervention Research, Rigshospitalet, Copenhagen University Hospital, Copenhagen, Denmark | [per.winkel@ctu.dk](mailto:per.winkel@ctu.dk) |
| Stefan Schwab | Department of Neurology, University Medical Centre Erlangen, Erlangen, Germany | Stefan.Schwab@uk-erlangen.de |
